# Anti-Glioma Activity Achieved by Dual Blood–Brain Barrier/Glioma Targeting Naive Chimeric Peptides-Based Co-Assembled Nanophototheranostics

**DOI:** 10.3390/pharmaceutics15010265

**Published:** 2023-01-12

**Authors:** Taru Dube, Jiban Jyoti Panda

**Affiliations:** Institute of Nano Science and Technology (INST), Mohali 160062, Punjab, India

**Keywords:** anti-glioma peptide, peptide drugs, co-assembly, peptide-based nanoparticles, combined chemo-phototherapy

## Abstract

Peptide monomers can either self-assemble with themselves enacting a solo-component assembly or they can co-assemble by interacting with other suitable partners to mediate peptide co-assembly. Peptide co-assemblies represent an innovative class of naive, multifunctional, bio-inspired supramolecular constructs that result in the production of nanostructures with widespread functional, structural, and chemical multiplicity. Herein, the co-assembly of novel chimeric peptides (conjugates of T7 (HAIYPRH)/t-Lyp-1 (CGNKRTR) peptides and aurein 1.2 (GLFDIIKKIAESF)) has been explored as a means to produce glioma theranostics exhibiting combinatorial chemo-phototherapy. Briefly, we have reported here the design and solid phase synthesis of a naive generation of twin-functional peptide drugs incorporating the blood–brain barrier (BBB) and glioma dual-targeting functionalities along with anti-glioma activity (G-Anti G and B-Anti G). Additionally, we have addressed their multicomponent co-assembly and explored their potential application as glioma drug delivery vehicles. Our naive peptide drug-based nanoparticles (NPs) successfully demonstrated a heightened glioma-specific delivery and anti-glioma activity. Multicomponent indocyanine green (ICG)-loaded peptide co-assembled NPs (PINPs: with a hydrodynamic size of 348 nm and a zeta-potential of 5 mV) showed enhanced anti-glioma responses in several cellular assays involving C6 cells. These included a mass demolition with no wound closure (i.e., a 100% cell destruction) and around 63% collaborative chemo-phototoxicity (with both a photothermal and photodynamic effect) after near infrared (NIR) 808 laser irradiation. The dual targeting ability of peptide bioconjugates towards both the BBB and glioma cells, presents new opportunities for designing tailored and better peptide-based nanostructures or nanophototheranostics for glioma.

## 1. Introduction

Amongst the significant hitches in glioma therapy, the prime one is to precisely parcel chemotherapeutics to the glioma cells across the blood–brain barrier (BBB). BBB levied limitations accompanied by the non-selective distribution and drug resistance offered by the brain tissues have stalled the process of achieving an effective glioma therapy [1]. Towards the headway and expansion of brain-targeting delivery systems, few nano-platforms with the aid of BBB-targeting ligands can be made to traverse through the BBB to directly reach the brain parenchyma. Disappointingly, only scant platforms with the ability to parcel loaded therapeutics to the diseased brain, after passing through the BBB, have been translated to clinics in a real sense. This demands the development of potent nanoneurotherapeutics that can precisely and accurately target both the BBB and glioma [2].

The supramolecular assembly of molecules into ordered structures represents one of the pronounced strategies to generate ensembles of supramolecular nanostructures with diverse biomedical applications. Among biomolecular self-assembly, peptides hold a unique place due to their many advents including the inherent potential shown by specific peptides to target and breach the BBB [2,3]. Peptide monomers can either self-assemble within themselves (i.e., solo-component assembly) or co-assemble by interacting with other suitable partners (i.e., peptide co-assembly). Co-assembly involves gathering more than two distinct peptides to form an ordered structure [4,5]. Peptide co-assemblies represent an innovative class of naive, multifunctional, bio-inspired supramolecular constructs that result in the production of nanostructures with widespread functional, structural, and chemical multiplicity [6]. Consequently, multicomponent peptide co-assembly has sprouted as an optimistic and expanded tactic with various advantages and has been efficiently exploited to fabricate novel nanoparticles (NPs) with customized and tailored features [4].

**Figure 1:**
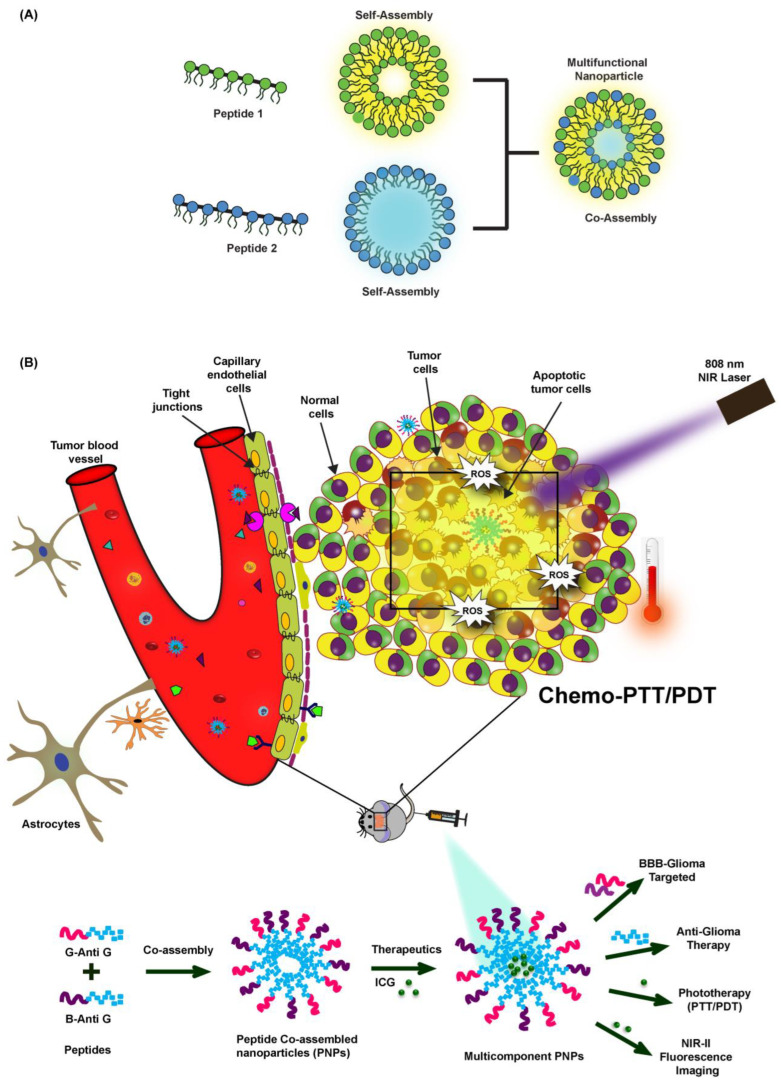
(**A**) Illustration showing peptide-based co-assembled multifunctional nanoparticles. (**B**) Scheme showing the synthesis of blood brain barrier (BBB)-glioma homing and anti-glioma chimeric peptides-based co-assembled nanoparticles (PNPs) loaded with indocyanine green (ICG) for combinatorial fluorescence-based imaging along with enabling the targeted chemo-phototherapy of glioma.

The incorporation of distinct peptide sequences into the co-assembly process synergistically imparts the constituent peptide’s characteristic features to the resulting structures (Figure 1A). Such multiple attributes, in many cases, are not feasible to be achieved from a solo component system (self-assembly). Additionally, the interplay among the mixed proportion of the distinct participating peptides enables one to tune the size, morphology, and other physical, chemical, and mechanical assets of the resultant structures [7]. The fascinating facet of self/co-assemblies is their dynamic feature owing to the non-covalent interactions covering hydrogen bonding, van der Waals, electrostatic, and stacking interactions. The overall assembly is ruled by the balance of these attractive and repulsive forces within and among the peptides. This makes the process simple and circumvents complicated synthesis, purification, and processing stages, thus producing flexible, self-healing, and stimuli-responsive structures [6,7].

Co-assembly can occur in four probable manners, that includes cooperative, self-sorting, random, and destructive assemblies. In cooperative assembly, peptides interact among themselves to produce structures comprised of both the components. A cooperative co-assembly induces multifunctionality. In orthogonal or self-sorted assembly, peptides assemble independently in the presence of each other, whereas in random assembly, the peptides organize without following any precise pattern. Alternatively, in destructive co-assembly, one of the partnering peptides acts as an inhibitor in the self-assembly procedure of the other and the process is very advantageous in regulating and tailoring the physical properties of the subsequent constructs [4,6].

Recently, Adler-Abramovich et al. have explored the co-assembly of fluorenyl methoxycarbonyl-diphenylalanine (Fmoc-Phe-Phe) and Fmoc-pentafluoro-phenylalanine (Fmoc-F5-Phe) peptides. Their co-assembly led to the fabrication of a highly rigid hydrogel, as compared to hydrogels self-assembled by Fmoc-Phe-Phe and Fmoc-F5-Phe on an individual level. The co-assembly dramatically enhanced the mechanical features of the resultant hybrid hydrogel [8]. In a similar attempt, Lynn et al. explored the co-assembly of two oppositely charged peptides. These peptides differed solely in their N-terminal amino acids residues, i.e., lysine (K) in the case of Ac-KLVFFAL-NH_2_ and phosphotyrosine (pY) in the case of Ac-(pY)LVFFAL-NH_2_. Their co-assembly resulted in the development of bilayer nanotubes with external negative and internal positive surfaces [9]. Similarly, Nilsson et al. studied the co-assembly of the L and D forms of Ac-(FKFE)_2_-NH_2_ and reported the development of rippled β-sheet fibrils analogous to amyloid fibrils. Unlike the self-assembled fibrils of L-peptides, the co-assembled structures were found to be more resistant to enzymatic degradation [10,11].

Here, via an effortless process, we have developed a naïve, multifunctional, theranostic nano-platform consisting of chimeric peptides-based co-assembled nanoparticles (PNPs) co-loaded with indocyanine green (ICG). We used these systems for anti-glioma drug delivery. The designed nanophototheranostics, upon near infrared (NIR) 808 irradiation aided in attaining a collaborative chemo-phototherapy (photodynamic therapy (PDT) and photothermal therapy (PTT)) coupled with fluorescence-based cell imaging (Figure 1B). Excellent BBB-glioma dual targeting and anti-glioma activity was realized via these novel, chimeric peptides-based nanoformulations.

## 2. Materials and Methods

### 2.1. Materials

All amino acids, 1,1,1,3,3,3-hexa-fluoro-2-propanol (HFIP), rhodamine 6G (Rho), Hoechst 33342 were procured from Merck Sigma-Aldrich (Munich, Germany). Dulbecco’s modified eagle medium (DMEM), antibiotic, and trypsin were purchased from Gibco, ThermoFisher Scientific Inc., (New York, USA). All the materials were handled as received and their respective aqueous solutions were prepared in deionized water (DI). Further details are provided in the Supplementary Materials.

### 2.2. Solid Phase Synthesis of Chimeric Peptides with Anti-Glioma Activity

Chimeric peptide drugs, namely, G-Anti G (CGNKRTRGGGLFDIIKKIAESF) and B-Anti G (HAIYPRHGGGLFDIIKKIAESF), were synthesized in a fully automated and microwave-aided Liberty Blue CEM peptide synthesizer (Matthews, NC, USA) at a scale of 0.10 mmol and were characterized using high-performance liquid chromatography (HPLC) and mass spectrometry (Waters, Milford, MA, USA) as reported earlier by our group [12,13]. The peptides were also evaluated for their glioma homing/specificity and anti-glioma properties in glioma as well as non-glioma cell lines [12,13]. The details are provided in the Supplementary Materials.

### 2.3. Synthesis of Multicomponent Peptide Co-Assemblies

The chimeric peptide-derived co-assembled nanostructures (PNPs) were prepared following a protocol described previously by our group with slight variations [12,13,14,15]. A molar ratio of 1:1 for the G-Anti G and B-Anti G was chosen for the NPs preparation. The G-Anti G and B-Anti G (1 mg each) were mixed and dissolved in HFIP (50 µL), followed by bath sonication for ~10 min if required. Spontaneous co-assembly into the nanostructures was triggered by the rapid inclusion of 950 µL of DI into the peptide mixture at room temperature (RT). The formed structures were aged for ~30 min before any experiment. Likewise, the peptide co-assembly was also studied by varying the solvent–cosolvent mixture. Self-assembly of the peptides was carried out at RT unless specified otherwise. The obtained aqueous dispersion of the peptide co-assemblies was ready-to-use and was stored at 4 °C for further usage.

### 2.4. Synthesis of ICG-Loaded PNPs

The ICG-loaded PNPs (PINPs) were prepared as follows. Briefly, evidently transpicuous stock solutions of the peptides (G-Anti G and B-Anti G, of 1 mg each) were prepared by dissolving lyophilized peptide powder in HFIP (50 µL). Then, ICG packaging into the NPs was instigated via the addition of an ICG solution (i.e., 0.1 mg in 950 µL of an acetate buffer) to the peptide mixture. The co-assembled PINPs were then incubated with constant shaking at RT (at 50 rpm, for 48 h). Afterward, the PINPs were ultracentrifuged (at 7000 rpm, for 10 min, at 4 °C). The obtained PINPs were re-suspended in DI or a phosphate buffer (pH~7.4) for further usage.

### 2.5. Bio-Physical Characterization of PNPs

The hydrodynamic size distributions (Z. Avg.), polydispersity index (PDI), and zeta-potential measurements of the peptide nanostructures were performed using the dual angle dynamic light scattering (DLS) technique, which is also recognized as photon correlation spectroscopy (Zetasizer Nano ZSP; ZEN5600; Malvern, Worcestershire, UK). Briefly, 1 mL of NPs (0.1 mg/mL in DI) were taken into cuvettes (DTS0012; Malvern for size and DTS1070; Malvern for zeta) for the measurements. Electron microscopic studies, i.e., scanning electron microscopy (SEM; JEM; JEOL; Tokyo, Japan) and transmission electron microscopy (TEM; JEM-2100; JEOL; Tokyo, Japan) were performed to additionally characterize and validate the morphology and dimensions of the peptide co-assemblies in greater detail. The encapsulation and loading efficiency of the ICG in the PINPs was determined by UV-visible spectroscopy. The concentration of unloaded ICG was assessed by recording its absorbance at 778 nm for the ICG using a double beam UV-visible spectrophotometer (Shimadzu UV-2600, Kyoto, Japan). The results were expressed as the entrapment and loading efficiency.

### 2.6. NIR-808 Laser Induced Temperature Rise by PINPs

The photothermal efficiency of the PNPs and PINPs (0–500 μg/mL) were studied using a NIR laser (Aimil Technologies, Delhi, India) as described previously [12,13]. The details are provided in the Supplementary Materials.

### 2.7. Cytotoxicity of PNPs Determined in C6 Cells

The viability and proliferation of C6 cells in the presence of PNPs was evaluated. In short, C6 cells (10,000/well in 96-well dishes) were seeded in quadruplicate and cultured in a complete growth media followed by 24 h incubation. Afterwards, they were treated with PNPs (i.e., 2.5, 5, 10, 15, 20, 25, and 30 µg/mL) and again incubated for 24 h, followed by a viability analysis using a standard MTT assay [12,13]. Finally, the optical absorption of the produced formazan at 570 nm was measured by a microtiter plate reader (Biotech Synergy H1, Espoo, Finland). Assuming the viability of the control untreated cells was 100%, the percentage of the cell viability was calculated as the percentage of the MTT reduction. The results are presented as median and quartiles (min to max) in quadruplicate. A statistical analysis was carried out using a Kruskal–Wallis test followed by a Dunn’s post hoc test.

### 2.8. Cellular Internalization of PNPs Determined in C6 Glioma Cells

For assessing the cellular uptake/internalization efficiency of the PNPs in C6 cells, PNPs were loaded with the fluorescent dye, rhodamine 6G (Rho). The cellular internalization of Rho-loaded PNPs as compared to free Rho was probed using a confocal microscope (LSM880 Carl Zeiss System, Munich, Germany). Concisely, C6 cells (50,000/well in 6-well dishes) were cultured in DMEM media as described above. Then, Rho-loaded PNPs (100 µg/mL) and a Rho solution were added to the respective wells and the sample was incubated further (until 2 h). Trailed to incubation, the nuclei were stained with Hoechst, followed by live imaging under a microscope (405 nm/Hoechst and 488 nm/Rho) [12,13]. The images were obtained and processed using the Zen blue LSM 880 software. The details are provided in the Supplementary Materials.

### 2.9. Localized Chemo-Phototoxicity of PINPs towards C6 Cells

The localized collaborative chemo-phototoxicity of PINPs, in contrast to PNPs, was studied and then visualized using C6 cells. In short, to assess the thermal ablation effect, C6 cells (50,000/well in 6-well dishes) were seeded and cultured in complete media and they were treated with different formulations (100 μg/mL, for 30 min). The used complete growth media containing the formulations was then swapped with new phenol-free media without FBS. For a combined chemo-phototoxicity analysis, the corresponding wells containing cells were exposed to a NIR-808 laser for ~10 min, incubated for an additional 30 min, and subsequently imaged under a brightfield microscope (Olympus BX53; Tokyo, Japan) [12,13].

### 2.10. Scratch Wound Healing Assay for Determining the Effect of PINPs on C6 Glioma Cell Migration

Scratch wound healing assays of the PNPs, PINPs, and ICG (100 μg/mL) were studied in C6 cells as described previously [12,13]. This was followed by imaging of the cells which migrated towards the cell-free zone in the scrape at 0 and 24 h, using a brightfield inverted microscope (IX71; Olympus Corp., Tokyo, Japan). The cell migration was analyzed by measuring the dimension of the gap distance using the Adobe Photoshop CC software. The details of the process being used are provided in the Supplementary Materials.

### 2.11. Determination of Localized Chemo-Phototoxicity of PINPs in C6 Cells

Next, we quantified the synergistic chemo-phototoxicity of PINPs (10 μg/mL) in comparison to other individual moieties (PNPs (chemotherapy) and ICG (phototherapy)) in the C6 cells as described previously [12,13]. The results are presented as the median and quartiles (min to max) in quadruplicate. A statistical analysis was carried out using a Kruskal–Wallis test followed by a Dunn’s post hoc test for a pair-wise comparison between the different groups. A p value less than 0.05 was considered to be statistically significant. The details are provided in the Supplementary Materials.

## 3. Results and Discussion

### 3.1. Chimeric Therapeutic Peptides

Like other tumor cells, glioma cells overexpress a unique set of proteins/receptors on their surfaces [16]. Judiciously targeting glioma-specific proteins/receptors presents the opportunity of precisely fabricating glioma-targeted delivery systems [17]. Quite a significant amount of BBB and glioma-specific ligands are being reported, including proteins/peptides/polypeptides, antibodies or their fragments, and others [18,19]. Glioma-targeting peptides serve as an efficient alternative for exactly and precisely targeting the glioma-specific proteins/receptors. As compared to other ligands, glioma-specific peptides exhibit a heightened diffusivity and offer trouble-free synthesis/chemical amendment procedures to further improvise their stability and overall pharmacokinetics [18]. These peptide ligands can be used as glioma-targeted theranostics. Additionally, these peptide ligands can be further engineered to have inherent anti-cancer activity, thereby inhibiting tumor progression and spread [20].

Hence, peptides can serve as promising and new-fangled therapeutic candidates for glioma with the potential for more effective and precise targeting with minimal adverse events compared to the conventional onco-therapies. Owing to their ability to aim at the proteins/receptors which are exclusively overexpressed on glioma cells or precisely aiming dysregulated signaling pathways, peptides exhibit a greater probability for curing even the most aggressive tumors such as glioblastoma. One approach to further expand the specificity and selectivity of peptides-based theranostics is to amalgamate them with other onco-homing peptides [21]. Peptides, after locking with their specific targets, can be used to deliver drugs and imaging agents, to destroy cancer cells, and to serve as antagonists to various ligands [18]. They offer additional medicament choices alone or in combinatorial therapy [22].

While peptide-mediated, site-specific drug delivery intensifies the practicality of onco-therapy with minimal detrimental effects in nearby healthy tissues, they still suffer from certain limitations, such as instability in the circulatory system and poor in vivo bioavailability. Chemical modification or amalgamation/coupling with macromolecules and nanocarriers will improve the stability of peptides by making them resistant to proteolytic degradation.

In one of our recent works, we synthesized a naive category of dual-functional peptide drugs, namely, the G-Anti G and B-Anti G, and experimentally demonstrated their BBB/glioma-targeting abilities along with a chemo PDT-PTT effect by individually amalgamating them with peptide/peptide-metal NPs (i.e., G-Anti G with poly(levodopamine) and B-Anti G with gold nanoroses). They demonstrated enhanced combinatorial chemo-phototherapy in all the efficacy studies carried out in C6 glioma cells and spheroids combined with the anti-cancer drug doxorubicin (DOX) [12,13]. Here, we tried to further extend our library of peptide-based NPs via addressing their multicomponent co-assembly and by testing their potency in glioma cells. Briefly, in G-Anti G, a BBB-glioma homing peptide (tLyp-1) was coupled to aurein 1.2, an anti-glioma peptide with a GG linker to form an entirely naive sequence (CGNKRTRGGGLFDIIKKIAESF), and in B-Anti G, a BBB-glioma homing peptide (T7) was coupled to aurein 1.2 peptide with a GG linker to form another naive sequence (HAIYPRHGGGLFDIIKKIAESF). Aurein 1.2 is an amphipathic cationic peptide (GLFDIIKKIAESF) with both anti-cancer and antimicrobial activities and it facilitates the disintegration of the plasma membranes, trailed by killing via a toroidal pore model [12,13,23]. These new peptide sequences were documented to be suitable to function as competent multi-targeting ligands to augment their efficacy against glioma by simultaneously dispatching chemotherapeutics across the BBB in mice and to glioma cells, along with acting as anti-glioma drugs [12,13].

### 3.2. Fabrication and Bio-Physical Characterization of the Multicomponent Peptide Co-Assembled Nanoparticles (PNPs)

Self-assembling peptides are bioactive and have been shown to self-assemble into an array of nano/microstructures, covering hydrogels, fibrils/fibers, rods/tubes, necklaces, films/sheets, leaflets, plates, nanocages, spheres, vesicles, etc. The resultant structures from these advanced materials can have countless applications in nanomedicine and related fields, such as tissue engineering, regeneration, diagnostics, and drug/gene delivery [24,25]. In this regard, we highlight our stratagem of utilizing a molecular self-assembly as a toolkit for generating co-assembled peptide-based nanoplatforms as potential glioma theranostics.

The co-assembly of multiple peptides offers a promising method for fabricating novel nanostructures with amplified and synergistic activities. Venturing forward in this direction, we co-assembled two different peptides in HFIP-water at a physiological pH, with each bearing a different targeting ligand conjugated to an anti-glioma peptide. We anticipated that a co-assembly would lead to the fabrication of mixed nanostructures that simultaneously could present two targeting ligands on the exterior/surface of the nanostructures along with the ability of killing the glioma cells, thus holding a high potential in the multi-targeted therapy against glioma.

Based on the results obtained by the self-assembly of the individual peptides (Figure S1, Supplementary Materials), a molar ratio of 1:1 G-Anti G/B-Anti G was chosen for achieving the peptide co-assembly. A DLS analysis revealed that the chimeric peptides (G-Anti G and B-Anti G) at a molar ratio of 1:1, co-assembled in HFIP-water (1:19 *v*/*v*) to form discrete structures with a mean diameter of 348 nm (PDI: 0.5) and a zeta-potential of 5 mV. The size and morphology of the PNPs determined using SEM pointed towards the formation of fibrillar structures and some spherical nuclei-like morphologies with an average diameter of 240 nm (Figure S2, Supplementary Materials).

### 3.3. Determination of the Outcome of Different Assembling Conditions on the Peptide Co-Assembly

Researchers working in the arena of self-assembled peptide-based nanostructures have also tried to interpret the effect/outcome of different assembling parameters on the final properties of the so-formed nanostructures. Innumerable parameters (such as concentration, solvent polarity, ionic strength, pH change, temperature, etc.) significantly influence the characteristic features of the peptide NPs [24,26,27]. Therefore, to explore this, we further examined the consequences of different parameters on the co-assembling behavior of the peptides.

#### 3.3.1. Effect of the Dispersing Solvent on the Peptide Co-Assembly

The solvent polarity and dielectric constant play an imperative role in governing the morphology, size, and other properties of the self-assembled nanostructures. Alterations in the solvent media for assembly represent a simple and appropriate tactic to govern the structural and morphological features of peptide-based co/self-assemblies and to fabricate customized NPs tailored for specific requirements [28,29,30,31,32]. Hence, the effect or outcome of diversified solvents on the co-assembly between G-Anti G and B-Anti G was further inquired in other organic solvents, as few solvents may be favored over others for their probable use in cells or biological systems.

Herein, we picked up various dispersing solvents such as ethanol, dimethyl sulfoxide (DMSO), isopropanol (IP), and acetone and tried to co-assemble the peptides at a solvent to water ratio of 1:19 *v*/*v*. It was noted that analogous to HFIP, the peptides were also proficient in adopting distinct morphological structures in various dispersing solvents. The mean hydrodynamic diameter and PDI of PNPs in the various solvents are presented in Table 1. The results revealed the formation of nanostructures with a diameter of 245 nm (PDI: 0.4) in HFIP, 348 nm (PDI: 0.5) in DMSO, 382 nm (PDI: 0.4) in ethanol, 456 nm (0.4) in IP, and 306 nm (PDI: 0.5) in acetone, and they demonstrated that the overall size and the monodispersity of the peptide-based nanostructures can be governed/ruled and controlled by judiciously selecting the assembling conditions.

Furthermore, to understand the overall morphological features and the dimensions of peptides-based co-assemblies in different solvents, SEM was carried out. It was noted that the G-Anti G and B-Anti G co-assembly resulted in the formation of microfibrillar aggregates in the HFIP, and small particulate structures in the ethanol, DMSO, and IP, whereas no specific morphology was obtained in the case of acetone as the initial dispersing solvent. The peptides demonstrated a solvent-dependent co-assembly similar to other peptides/amino acid mimetics.

Keeping in view the hands-on applications in glioma drug delivery, small sized peptide NPs (≤300 nm) with a monodisperse size distribution are the choice-worthy candidates, because smaller NPs prevail for a longer period in the circulation, and subsequently having a greater likelihood of passively accumulating in glioma, whereas uniformity ensures reproducibility in delivering precise quantities of the encapsulated chemotherapeutics per dose. Based on the DLS and SEM analysis, HFIP was preferred as the preliminary dispersing solvent for enabling chimeric peptide co-assembly for further studies.

#### 3.3.2. Effect of Ions/Salts on the Peptide Co-Assembly

Ions have also been manifested to influence and govern molecular self-assembly. In fact, the salt concentration has been known to alter or fluctuate the overall ionic strength of the solutions, which could not only initiate the process of molecular co/self-assembly by forming intermolecular bridges but also has been shown to alter the overall process, eventually leading to the fabrication of nanostructures with variable properties [33,34,35]. An earlier effort via Hong et al. illustrated the effect of sodium chloride (NaCl) on the self-assembly behavior of the AEAEAKAKAEAEAKAK (EAK16-II) [33]. Additionally, an earlier work by Panda et al. illustrated a conformation shift in phenylalanine-α,β-dehydrophenylalanine (FΔF)-based hydrogels fabricated in varying sodium acetate concentrations via a circular dichroism analysis [36].

We anticipated that the PNPs may have exhibited a salt-dependent assembly behavior and would exhibit morphological perturbations with the variations in the ionic strength. Therefore, we attempted to study our peptide co-assembly by varying the final assembling conditions from water to other aqueous buffers such as phosphate buffer saline (PBS), 4-(2-hydroxyethyl)-1-piperazineethanesulfonic acid (HEPES), 0.2 M acetate, and 0.1% NaCl, while keeping the initial dispersing solvent (HFIP) fixed. Hence, we endeavored to explore other more suitable solvent combinations (e.g., HFIP–PBS, HFIP–HEPES, HFIP–acetate, HFIP–NaCl, etc.) for achieving an efficient peptide co-assembly apart from the HFIP–water pair. The average diameter and PDI of the peptides-based co-assemblies in the HFIP–buffer mixtures is presented in Table 2. It was noticed that except for the combination of HFIP with an acetate buffer, the obtained size and polydispersity of the co-assembled NPs was inappropriate for brain drug delivery applications. The peptides co-assembled into nanostructures had an average size of 1175 nm (PDI: 0.3) in a mixture of HFIP–HEPES, 337 nm (PDI: 0.1) in a mixture of HFIP–acetate, and 1077 nm (PDI: 0.3) in HFIP–NaCl.

These studies indicated that the assembling conditions played a distinguished part in deciding the end properties or functionalities of the resultant co/self-assembled structures, including the size, morphology, and polydispersity.

**Figure 2:**
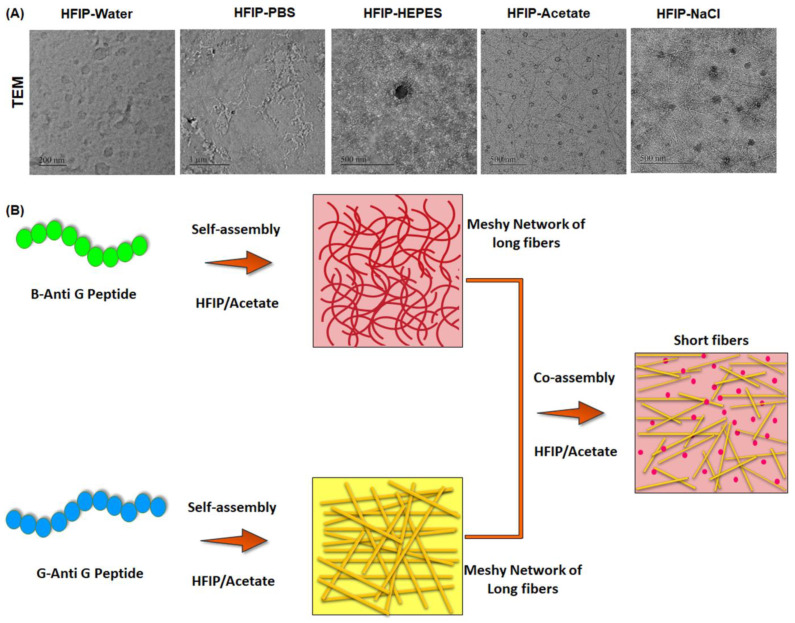
(**A**) Transmission electron micrographs of peptide-based co-assembled nanostructures (PNPs) formed in various HFIP–buffer mixtures. (**B**) Illustration representing peptide co-assemblies as an innovative class of naive, multifunctional, bio-inspired supramolecular constructs.

Electron microscopy was carried out to understand the final morphological features of the peptides-based co-assemblies formed in different HFIP–buffer mixtures. The SEM micrographs reflected the existence of dual morphologies in few cases. It was observed that the G-Anti G and B-Anti G co-assembly resulted in the formation of both spherical and fibrous structures in the HFIP–water, HFIP–PBS, HFIP–acetate, and HFIP–NaCl mixtures, whereas, the co-assembly of the peptides led to the formation of NPs in the case of the HFIP–HEPES. Similarly, the TEM micrographs (Figure 2A) pointed towards the presence of spherical NPs in the HFIP–water, HFIP–PBS, and HFIP–HEPES, whereas, spherical NPs and small fibers in the HFIP–acetate, and spherical NPs and large fibers in the HFIP–NaCl were obtained. Co-assembly dramatically reduced the size of the resultant hybrid peptide-based nanostructures. Based on the characterization, and with the aim to achieve potential glioma theranostics, peptide co-assemblies in the HFIP–acetate were selected and further efficacy studies were then carried out with them.

### 3.4. Cytotoxicity of PNPs Determined in C6 Cells

Next, we investigated the cytotoxicity of PNPs against C6 cells. As verified by the standard MTT assay (Figure S3, Supplementary Materials), it was noted that with increasing the concentration of PNPs, a decline in the proliferation rate of the C6 cells was observed, thereby resulting in decreased cell viability.

This PNPs-based cytotoxicity could be ascribed to the presence of aurein 1.2 (anti-glioma peptide), as one of the motifs of the chimeric construct. Furthermore, it was noted that at 30 µg/mL, the PNPs were highly cytotoxic (i.e., a 91% cell killing); however, at lower concentrations (2.5 µg/mL) a minor percentage of cell death was obtained (i.e., a 22% cell killing). These findings demonstrated that at lower concentrations, PNPs can be used as precisely-targeted glioma drug delivery nanosystems.

### 3.5. Synthesis of ICG-Loaded PNPs (PINPs)

Peptides can be rationally designed as excellent carriers for the delivery and transportation of multifarious anti-cancer therapeutics, which includes tiny molecular drugs (e.g., DOX, temozolomide, paclitaxel, etc.), oligonucleotides (e.g., aptamers, DNA, siRNA, etc.), radionuclides, imaging agents, cytotoxic peptides, and others [24,25].

Here, a combinatorial approach was outlined in which the PNPs were loaded with the NIR-dye ICG in an attempt to leverage them with a PTT/PDT effect and NIR-fluorescence-based bioimaging. ICG can be used as a photosensitizer and as a photothermal transducer to attain concurrent photothermal and photodynamic effects. Moreover, several studies have shown that the amalgamation of phototherapy with chemotherapy increases the vulnerability of cancer cells towards chemotherapeutics, thus producing better synergistic anti-cancer results. Consequently, it is expected that ICG-mediated PTT/PDT would synergistically amplify the therapeutic properties of the synthesized PNPs. ICG was successfully loaded onto the peptide co-assemblies (PINPs), and the packaging of ICG was verified by UV-vis spectroscopy. The ICG encapsulation and loading efficiencies were calculated to be ~100% and 3%, respectively.

### 3.6. NIR-808 Laser-Induced Temperature Rise Achieved by the PINPs

**Figure 3:**
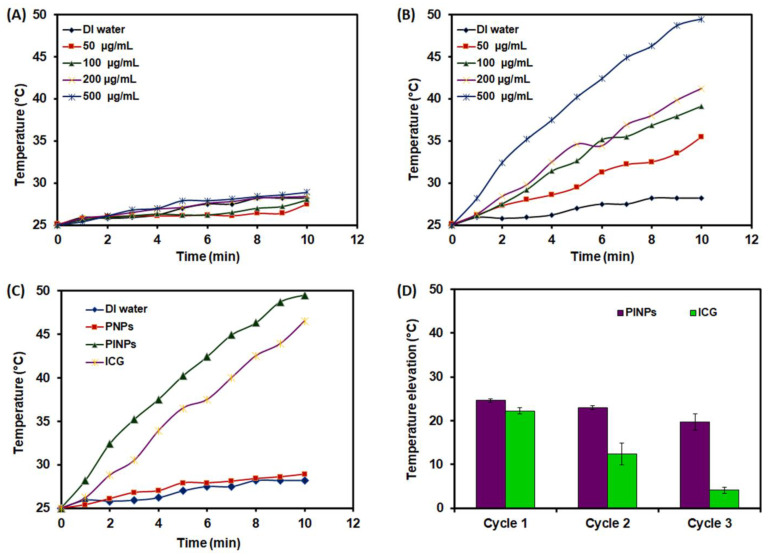
Photothermal effect of PNP and PINPs. (**A**) Temperature rising graphs of PNPs determined at various concentrations under NIR-808 exposure (2 W/cm^2^) and compared against DI water. (**B**) Temperature rising graphs of PINPs determined at various concentrations under NIR-808 laser and compared against DI water. (**C**) Temperature rising graphs of DI water, PNPs, PINPs, and ICG determined at similar concentration of PNPs or ICG. (**D**) Temperature rising graphs of PINPs and ICG determined at similar ICG concentrations with three successive on/off rounds of NIR-808 exposure. Results are presented as mean ± SD in triplicate.

The laser-disposed temperature elevation of the PNPs and PINPs was probed under NIR-808 laser irradiation (Figure 3). The local hyperthermia was recorded in order to estimate the PTT effect. Figure 3A shows the ICG-based temperature elevation graphs of PNPs under NIR-808 laser irradiation at several concentrations (50–500 µg/mL). It was observed that on increasing both the irradiation period and the PNPs concentration, no obvious escalation in the temperature of the PNPs aqueous dispersion was perceived in comparison to that achieved in the case of DI under the same settings. Figure 3B shows the temperature elevation curves of the PINPs obtained at various concentrations. In the PINPs, it was observed that the temperature escalated on increasing both the irradiation period and the PINPs concentration. Subsequently, after 10 min of irradiation, the temperature of the PINPs escalated to 36, 39, 41, and 50 °C at different concentrations, respectively. Figure 3C shows the temperature elevation graphs of the PINPs in comparison to the DI, PNPs, and ICG. At ICG concentration of 27 μg/mL, and after 10 min of laser irradiation, the temperature of the PINPs got escalated to 50 °C in comparison to the DI and ICG (46 °C). The obtained results validated the photothermal energy-transducing efficiency of the synthesized PINPs.

It was also noted that the PINPs further exhibited a higher photo-stability as opposed to the free ICG solution. Temperature elevations (Figure 3D) of 25, 23, and 20 °C for the PINPs and 22, 12, and 4 °C for the native ICG were witnessed after three successive on/off cycles of the NIR-808 laser, respectively.

### 3.7. Cellular Internalization of PINPs in C6 Cells

**Figure 4:**
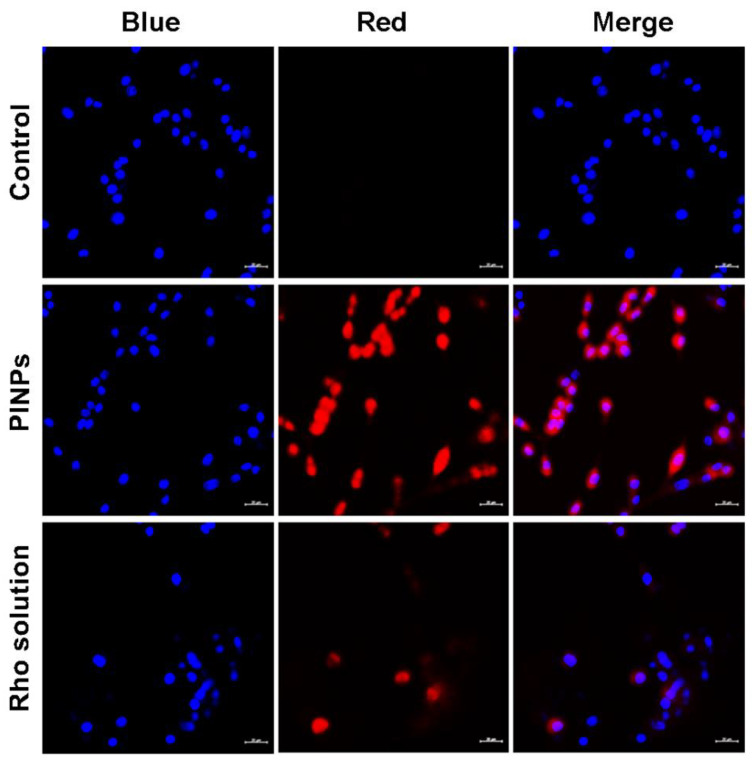
Confocal micrographs depicting cellular internalization of rhodamine 6G (Rho)-loaded PINPs after 2 h of co-incubation with C6 cells. Blue color: Hoechst (staining nucleus); Red color: Rho (staining cytoplasm). Scale bar: 20 µm.

For realizing a successful glioma therapy, the effective delivery or transportation of therapeutics equally to a tumor core and infiltrative glioma cells is needed. Here, to effectuate precision-based glioma chemo/photo-theranostics, multifunctional peptide nanoplatforms have been fabricated by co-assembling bi-functional chimeric peptides with both a BBB and glioma-targeting ability, along with an anti-glioma functionality to act as active targeting nanophototheranostics. The glioma-targeting aptness of Rho-loaded PINPs in comparison to a Rho solution was investigated in vitro in C6 cells. Confocal laser scanning microscopic (CLSM) images (Figure 4) revealed that the Rho-loaded PINPs could effectively become internalized inside the cells, and they revealed a greater uptake in glioma cells as compared to the free Rho solution. The enhanced uptake of Rho-loaded PINPs may be attributed to the presence of glioma-targeting peptide sequences (i.e., a tLyp-1 peptide motif in G-Anti G and a T7 peptide motif in B-Anti G). These results confirmed the potency of PINPs as extremely efficient peptide-based nanophototheranostics for combating notorious gliomas.

### 3.8. Localized Chemo-PTT/PDT Toxicity of PINPs Determined in Glioma Cells

**Figure 5:**
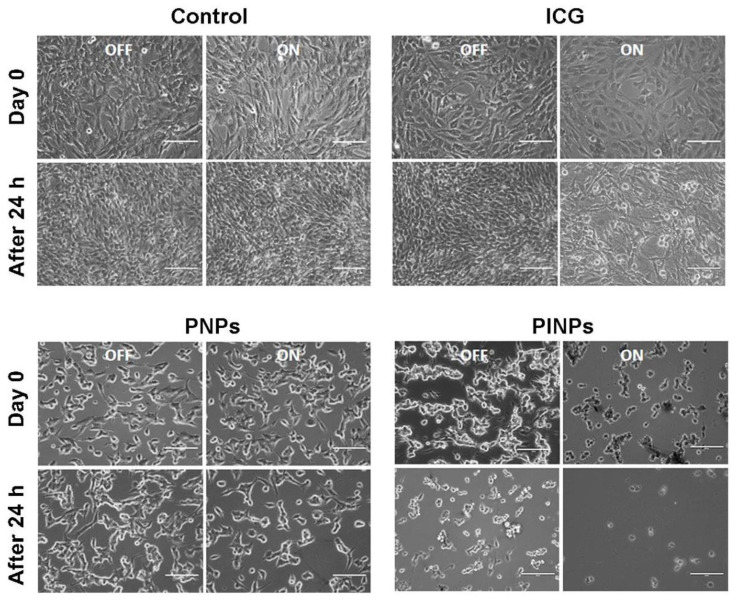
Brightfield images showing the collaborative chemo-PTT/PDT toxicity of PINPs exhibited in C6 cells. Scale bar: 100 µm.

Strengthened from the photothermal energy-transducing efficiency and higher glioma cell internalization of the PINPs, we further investigated their collaborative chemo-PTT/PDT toxicity. Brightfield pictures of the control cells with/without NIR-808 and ICG-treated cells with/without NIR-808 showed no cell death (Figure 5). This could be ascribed to the lower dose of the free ICG availed. Meanwhile, the PNPs-treated C6 cells kept in the dark and after NIR-808 exposure, and the PINPs-treated C6 cells kept in the dark, showed limited cell destruction. The observed cytotoxicity could be ascribed to the potential cell-killing effect of the anti-glioma peptide containing a motif derived from aurein 1.2. Furthermore, it was noted that the C6 cells incubated with PINPs after the NIR-808 exposure displayed a mass destruction as compared to those treated with PINPs but kept in the dark. It was noted that the cells treated with PINPs in the presence of a laser exposure exhibited ~100% cell death following 24 h of incubation. Thus, the combined chemo-photokilling of the ICG and PNPs group was significantly higher as compared to only the PNPs-treated cells. This can be ascribed to the combination of targeted and anti-glioma toxicity effects mediated by the peptides (i.e., G-Anti G/B-Anti G) and the PDT/PTT effect mediated by the ICG. The results advocate that the packaging of ICG onto PNPs would improve its phototherapeutic efficacy and could successfully destroy glioma cells even at a very small dosage of the individual therapeutic modalities. The multifunctional nano-platform, i.e., the PINPs, was proved to be very effective in realizing a collaborative chemo-PTT/PDT effect.

### 3.9. Scratch Wound Healing Assay for Determining the Effect of PINPs on C6 Glioma Cell Migration

**Figure 6:**
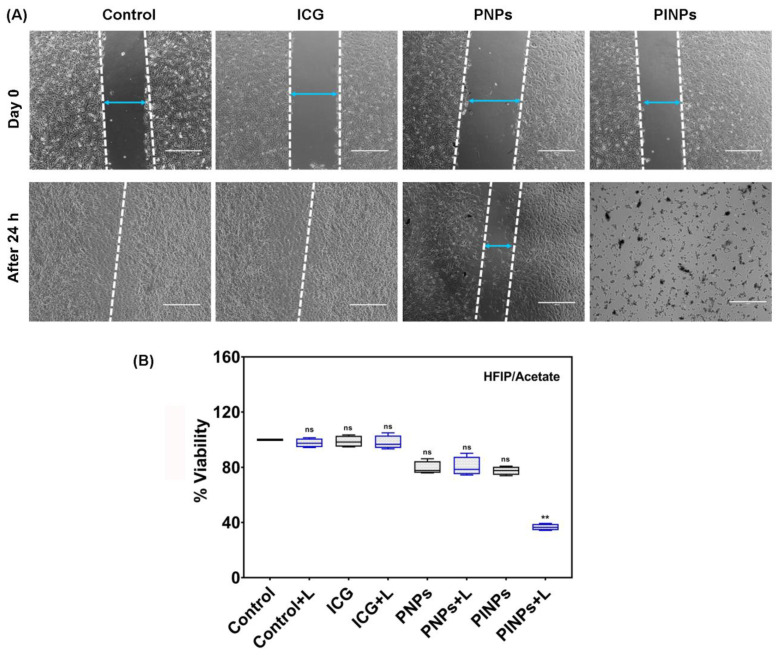
(**A**) Scratch assay showing migration of C6 cells after being incubated with PINPs, ICG, PNPs, and control untreated cells after laser irradiation. The dotted lines mark the areas lacking cells and blue color arrows represents the gap width. (magnification: ×400). (**B**) Collaborative chemo-PTT/PDT toxicity of PINPs (5 µg/mL) in C6 cells before and after laser irradiation. Results are presented as median and quartiles (min. to max.) in quadruplicate. The bar inside the box represents the median. Statistical analysis was carried out using a Kruskal–Wallis test followed by a Dunn’s post hoc test. Asterisk (*) refers to the statistically significant difference between the control untreated cells vs. other groups. (** *p* < 0.01; ns = non-significant).

Next, we carried out a scratch assay to investigate the potency of the PINPs towards inhibiting the migration of C6 glioma cells. As depicted in Figure 6A, the control untreated and the ICG-treated cells successfully drifted across the scratch, past 24 h of scrapping, exhibiting ~100% wound closure after laser irradiation. The results pointed towards a weak phototherapeutic effect of the ICG at a small dosage and further validated our previous phototoxicity data. In contrast, the PINPs-treated cells after a NIR-808 exposure demonstrated no wound closure with 100% cell destruction. Such a mass demolition of cells post 24 h of irradiation will help in boosting the efficiency of the engineered nano-platforms and curtailing the relapse rates linked with glioma and other cancerous tissues.

Thus, the PINPs demonstrated a fruitful repression towards a migratory effect of glioma cells and can be possibly utilized as nanophototheranostic agents for achieving anti-glioma therapy.

### 3.10. Collaborative Chemo-PTT/PDT Toxicity of PINPs Determined in Glioma Cells

Next, we quantitatively measured the collaborative chemo-PTT/PDT toxicity of PINPs in C6 cells using a MTT assay. It was observed that upon a NIR-808 exposure, the cell viability declined significantly in the presence of PINPs (5 μg/mL; Figure 6B). As anticipated, it was noted that the cells incubated with PINPs after the NIR-808 irradiation showed higher killing (with a ~63% cell destruction) as compared to the PINPs in the dark (with a ~22% cell destruction). This remarkably heightened the therapeutic efficacy of the PINPs, and could be credited to the summation of a targeted and anti-glioma effect emanating from the therapeutic peptides, namely, G-Anti G/B-Anti G, and the PTT/PDT effect emanating from the ICG, with both culminating in an enhanced chemotherapy. The cell viability of the groups exposed to the collaborative chemo-PTT/PDT therapy was found to be the least as compared to those cells exposed to individual single moieties such as phototherapy and chemotherapy alone. These results again were directed towards the manifestation of collective effects when a targeted chemotherapy was combined with phototherapy. These findings demonstrated that PINPs hold great promise as effective multimodal nanophototheranostics for in vivo glioma therapy.

Multicomponent indocyanine green (ICG)-loaded peptide co-assembled NPs (i.e., PINPs: with a hydrodynamic size of 348 nm and a zeta-potential of 5 mV) showed enhanced anti-glioma responses in several cellular assays involving C6 cells. These included a mass demolition with no wound closure (100% cell destruction and around 63% collaborative chemo-phototoxicity).

## 4. Conclusions

In this report, we have fabricated dual functional, fluorescent, and ICG-loaded chimeric therapeutic peptides co-assembled NPs (PINPs), a potentially beneficial nanoplatform to enable a heightened and synchronized fluorescence imaging and targeted chemo-phototherapy in glioma. Multicomponent PINPs showed a feasible size (348 nm) and polydispersity for brain drug delivery. Their combinatorial chemo-PTT/PDT effect was probed in C6 cells. They showed enhanced anti-glioma responses in several cellular assays including a mass demolition with no wound closure (with 100% cell destruction) and around 63% collaborative chemo-phototoxicity. Compared to free, discrete therapeutic moieties, the engineered nano-platforms, namely, the PINPs, manifested a remarkably higher collective chemo-PDT/PTT efficacy. In total, these novel peptides-based nanophototheranostic systems represent a multifaceted combinatorial armament encompassing a dual glioma and BBB-targeting ability, and PTT/PDT activity along with combined chemotherapy that is achieved by means of designed anti-glioma peptide drugs. In conclusion, such a peptide-based glioma combotherapeutic modality will offer new opportunities in designing specific, peptide-based nanosystems for targeted glioma theranostics.

## Figures and Tables

**Table 1:** Table showing size and PDI of PNPs formed in various solvent-water (1:19 *v*/*v*) mixtures.

Peptide	Dispersing Solvent	Assembling Solvent	Z. Avg. (nm)	PDI
G-Anti G And B-Anti G	HFIP	Water	245	0.4
	DMSO	Water	348	0.5
	Ethanol	Water	382	0.4
	IP	Water	456	0.4
	Acetone	Water	306	0.5

PDI: polydispersity index; PNPs: chimeric peptide-derived co-assembled nanostructures; HFIP: 1,1,1,3,3,3-Hexa-fluoro-2-propanol; DMSO: dimethyl sulfoxide; IP: isopropanol.

**Table 2:** Table showing size and PDI of PNPs determined in various solvent-buffers (1:19 *v*/*v*) mixture.

Peptide	Dispersing Solvent	Assembling Solvent	Z. Avg. (nm)	PDI
G-Anti G And B-Anti G	HFIP	Water	348	0.5
		PBS	2.37 × 10^4^	0.8
		HEPES	1175	0.3
		Acetate	337	0.1
		0.1 NaCl	1077	0.3

PDI: polydispersity index; PNPs: chimeric peptide-derived co-assembled nanostructures; HFIP: 1,1,1,3,3,3-hexa-fluoro-2-propanol; PBS: phosphate buffer saline; HEPES: (4-(2-hydroxyethyl)-1-piperazineethanesulfonic acid); NaCl: sodium chloride.

## Data Availability

Not applicable.

## Supplementary Materials

The following supporting information can be downloaded at: https://www.mdpi.com/article/10.3390/pharmaceutics15010265/s1, Figure S1. Size distribution profile of self-assembled peptide nanostructures determined in a mixture of HFIP-water at a ratio of 1:19 *v*/*v*. Figure S2. Synthesis and characterization of multicomponent peptide based co-assembled nanostructures (PNPs) in HFIP-water (1:19 *v*/*v*) mixture. (A) Size distribution profile. (B) Scanning electron microscopic image of G-Anti G and B-Anti G co-assembled nanoparticles. Figure S3. Dose-response experiment i.e., cell viability of C6 glioma cells determined after 24 h of incubation with increased concentrations of PNPs (assembled in HFIP-acetate mixture). Results are presented as median & quartiles (min to max) in quadruplicate. The bar inside the box represents the median. Statistical analysis was carried out using Kruskal–Wallis test followed by Dunn’s post hoc test. Asterisk (*) refers to the statistically significant difference between the control untreated cells vs other concentrations. (* *p* < 0.05; ** *p* < 0.01; ns = non-significant).

## References

[1] Shergalis A., Bankhead A., Luesakul U., Muangsin N., Neamati N. (2018). Current challenges and opportunities in treating glioblastoma. Pharmacol. Rev..

[2] Gu G., Gao X., Hu Q., Kang T., Liu Z., Jiang M., Miao D., Song Q., Yao L., Tu Y. (2013). The influence of the penetrating peptide iRGD on the effect of paclitaxel-loaded MT1-AF7p-conjugated nanoparticles on glioma cells. Biomaterials.

[3] Wanjale M.V., Kumar G.S.V. (2017). Peptides as a therapeutic avenue for nanocarrier-aided targeting of glioma. Expert Opin. Drug Deliv..

[4] Makam P., Gazit E. (2018). Minimalistic peptide supramolecular co-assembly: Expanding the conformational space for nanotechnology. Chem. Soc. Rev..

[5] Xu Z., Jia S., Wang W., Yuan Z., Jan Ravoo B., Guo D.S. (2019). Heteromultivalent peptide recognition by co-assembly of cyclodextrin and calixarene amphiphiles enables inhibition of amyloid fibrillation. Nat. Chem..

[6] Fleming S., Debnath S., Frederix P.W.J.M., Hunt N.T., Ulijn R.V. (2014). Insights into the coassembly of hydrogelators and surfactants based on aromatic peptide amphiphiles. Biomacromolecules.

[7] Aida T., Meijer E.W., Stupp S.I. (2012). Functional supramolecular polymers. Science.

[8] Halperin-Sternfeld M., Ghosh M., Sevostianov R., Grigoriants I., Adler-Abramovich L. (2017). Molecular co-assembly as a strategy for synergistic improvement of the mechanical properties of hydrogels. Chem. Commun..

[9] Li S., Mehta A.K., Sidorov A.N., Orlando T.M., Jiang Z., Anthony N.R., Lynn D.G. (2016). Design of asymmetric peptide bilayer membranes. J. Am. Chem. Soc..

[10] Swanekamp R.J., DiMaio J.T., Bowerman C.J., Nilsson B.L. (2012). Coassembly of enantiomeric amphipathic peptides into amyloid-inspired rippled β-sheet fibrils. J. Am. Chem. Soc..

[11] Swanekamp R.J., Welch J.J., Nilsson B.L. (2014). Proteolytic stability of amphipathic peptide hydrogels composed of self-assembled pleated β-sheet or coassembled rippled β-sheet fibrils. Chem. Commun..

[12] Dube T., Kumar N., Bishnoi M., Panda J.J. (2021). Dual blood–brain barrier–glioma targeting peptide–poly(levodopamine) hybrid nanoplatforms as potential near infrared phototheranostic agents in glioblastoma. Bioconjugate Chem..

[13] Dube T., Kompella U.B., Panda J.J. (2022). Near infrared triggered chemo-PTT-PDT effect mediated by glioma directed twin functional-chimeric peptide-decorated gold nanoroses. J. Photochem. Photobiol. B Biol..

[14] Dube T., Mandal S., Panda J.J. (2017). Nanoparticles generated from a tryptophan derivative: Physical characterization and anti-cancer drug delivery. Amino Acids.

[15] Dube T., Kumar N., Kour A., Mishra J., Singh M., Prakash B., Panda J.J. (2019). Gold nano-/microroses on levodopa microtubes for SERS-based sensing of gliomas. ACS Appl. Nano Mater..

[16] Sharma P., Debinski W. (2018). Receptor-targeted glial brain tumor therapies. Int. J. Mol. Sci..

[17] Jones A.R., Shusta E.V. (2007). Blood-brain barrier transport of therapeutics via receptor-mediation. Pharm. Res..

[18] Oller-Salvia B., Sánchez-Navarro M., Giralt E., Teixidó M. (2016). Blood–brain barrier shuttle peptides: An emerging paradigm for brain delivery. Chem. Soc. Rev..

[19] Wang S., Meng Y., Li C., Qian M., Huang R. (2015). Receptor-mediated drug delivery systems targeting to glioma. Nanomaterials.

[20] Hoskin D.W., Ramamoorthy A. (2008). Studies on anticancer activities of antimicrobial peptides. Biochim. Biophys. Acta.

[21] Ellerby H.M., Arap W., Ellerby L.M., Kain R., Andrusiak R., Rio G.D., Krajewski S., Lombardo C.R., Rao R., Ruoslahti E. (1999). Anti-cancer activity of targeted pro-apoptotic peptides. Nat. Med..

[22] Raucher D. (2019). Tumor targeting peptides: Novel therapeutic strategies in glioblastoma. Curr. Opin. Pharmacol..

[23] Dennison S.R., Harris F., Phoenix D.A. (2007). The interactions of aurein 1.2 with cancer cell membranes. Biophys. Chem..

[24] Lee S., Trinh T.H.T., Yoo M., Shin J., Lee H., Kim J., Hwang E., Lim Y.-B., Ryou C. (2019). Self-assembling peptides and their application in the treatment of diseases. Int. J. Mol. Sci..

[25] Tesauro D., Accardo A., Diaferia C., Milano V., Guillon J., Ronga L., Rossi F. (2019). Peptide-based drug-delivery systems in biotechnological applications: Recent advances and perspectives. Molecules.

[26] Lowik D.W.P.M., Leunissen E.H.P., van den Heuvel M., Hansen M.B., van Hest J.C.M. (2010). Stimulus responsive peptide based materials. Chem. Soc. Rev..

[27] Ekiz M.S., Cinar G., Khalily M.A., Guler M.O. (2016). Self-assembled peptide nanostructures for functional materials. Nanotechnology.

[28] Kar S., Drew M.G., Pramanik A. (2011). Formation of vesicles through solvent assisted self-assembly of hydrophobic pentapeptides: Encapsulation and pH responsive release of dyes by the vesicles. Protein Pept. Lett..

[29] Ahmed S., Pramanik B., Sankar K.N.A., Srivastava A., Singha N., Dowari P., Srivastava A., Mohanta K., Debnath A., Das D. (2017). Solvent assisted tuning of morphology of a peptide-perylenediimide conjugate: Helical fibers to nano-rings and their differential semiconductivity. Sci. Rep..

[30] Rissanou A.N., Georgilis E., Kasotakis E., Mitraki A., Harmandaris V. (2013). Effect of solvent on the self-assembly of dialanine and diphenylalanine peptides. J. Phys. Chem. B.

[31] Fu I.W., Markegard C.B., Nguyen H.D. (2015). Solvent effects on kinetic mechanisms of self-assembly by peptide amphiphiles via molecular dynamics simulations. Langmuir.

[32] Mason T.O., Chirgadze D.Y., Levin A., Adler-Abramovich L., Gazit E., Knowles T.P.J., Buell A.K. (2014). Expanding the solvent chemical space for self-assembly of dipeptide nanostructures. ACS Nano.

[33] Hong Y., Pritzker M.D., Legge R.L., Chen P. (2005). Effect of NaCl and peptide concentration on the self-assembly of an ionic-complementary peptide EAK16-II. Colloids Surf. B Biointerfaces.

[34] Ozbas B., Kretsinger J., Rajagopal K., Schneider J.P., Pochan D.J. (2004). Salt-triggered peptide folding and consequent self-assembly into hydrogels with tunable modulus. Macromolecules.

[35] Sun J., Zhang H., Guo K., Yuan S. (2015). Self-assembly of dipeptide sodium salts derived from alanine: A molecular dynamics study. RSC Adv..

[36] Panda J.J., Mishra A., Basu A., Chauhan V.S. (2008). Stimuli responsive self-assembled hydrogel of a low molecular weight free dipeptide with potential for tunable drug delivery. Biomacromolecules.

