# Supplementary material for: Anti-Glioma Activity Achieved by Dual Blood–Brain Barrier/Glioma Targeting Naive Chimeric Peptides-Based Co-Assembled Nanophototheranostics
Source: Pharmaceutics. 2023 Jan 12;15(1):265. doi: 10.3390/pharmaceutics15010265 (PMC9863651; doi:10.3390/pharmaceutics15010265)
Supplement: Supplementary file 1 [file pharmaceutics-15-00265-s001.zip › pharmaceutics-2082896-supplementary.pdf]

pharmaceutics

MDPI

Supplementary Materials

Anti-Glioma Activity Achieved by Dual Blood–Brain Barrier/Glioma Targeting Naive Chimeric Peptides-Based Co-Assembled Nanophototheranostics

Taru Dube and Jiban Jyoti Panda

1. Materials and Methods

1.1. Materials

Ethanol, methanol, dichloromethane (DCM), dimethylformamide (DMF), dimethyl sulphoxide (DMSO), N,N′-diisopropylcarbodiimide (DIC), thioanisole, trifluoroacetic acid (TFA), 1,2-ethanedithiol (EDT), piperazine, oxyma, all Fmoc protected amino acids, Fmoc-Phe-Wang resin were obtained from Merck Sigma-Aldrich (Munich, Germany). Acetonitrile (HPLC grade), Isopropanol (IP), water (HPLC grade) and all other solvents utilized in the study were purchased from Merck Millipore (Darmstadt, Germany). Indocyanine green (ICG) was acquired from Tokyo Chemical Industry Co., Ltd. (TCI, China). Phosphate buffer saline (PBS), phenol free medium, fetal bovine serum (FBS), antibiotic and antimycotic mix-solution (penicillin: 100 U/mL; streptomycin: 100 µg/mL; and amphotericin B: 2.5 µg/mL), and trypsin EDTA solutions were purchased from Gibco, ThermoFisher Scientific Inc., (New York, USA). 3-(4,5-Dimethylthiazol-2-yl)-2,5-diphenyltetrazolium bromide (MTT) was acquired from HiMedia Laboratories Private Limited (India). All the aforementioned materials were used as received and the aqueous solutions were prepared with double filtered Milli-Q water (Ultrapure Water Purification Unit-Pure; Labocon, UK) with a resistivity of 18.2 MΩ cm^{-1}.

1.2. Cell Culture

Rat glioma (C6) cells cultured in DMEM in T25 flask was purchased from National Centre for Cell Science (NCCS), Pune, Maharashtra, India. After receiving, cells were maintained as monolayer cultures in DMEM enriched with FBS (10%). Further 1% of antibiotic and antimycotic mix-solution was added to the media (thereafter referred as complete growth media/complete media) to prevent any bacterial and fungal contamination during handling. For each study, cells were incubated inside a ThermoFisher Scientific CO_2 incubator upheld at 37 °C, 95% relative humidity, and 5% CO_2. For each experiment cells were first trypsinized (trypsin-EDTA solution in PBS) until 2-3 min as required and resuspended in complete growth media prior seeding. All assays were performed in quadruplet unless specified otherwise.

1.3. Statistical Analysis

Results were articulated as mean ± standard deviation (SD) or median & quartiles (min to max) as specified of at least four independent experiments. We have also performed a non-parametric test (Kruskal-Wallis) followed by Dunn’s post hoc test to establish significance between different groups in efficacy results. A p value less than 0.05 was considered to be statistically significant. Statistical analysis was performed with GraphPad Prism 9.0 software (GraphPad Software Inc., San Diego, CA, USA).

1.4. Methods

1.4.1. Solid Phase Synthesis of Chimeric Peptides (G-Anti G and B-Anti G) with Anti-Glioma Activity

Briefly, all essential Fmoc protected amino acids were weighed and dissolved in required volume of dimethylformamide (DMF) according to the scale of 0.10 mmol. Sidewise, Fmoc-Phe-Wang resin was pre-soaked in DMF for ~30 min to inflate the resin completely. During solid phase peptide synthesis, each time Fmoc deprotection from the amino acid was accomplished using 20% piperazine in DMF. Whereas, Fmoc-amino acid coupling was accomplished in DMF with microwave aided heating using diisopropylcarbodiimide (DIC, activator) and oxyma (activator base). A series of steps constituting coupling, deprotection, washing etc., were repeated until the desired chimeric peptide chain was synthesized. Finally, the resin anchored peptide was filtered and washed with DMF, dichloromethane (DCM), and methanol respectively until dried completely. Synthesized chimeric peptide sequence was then cleaved using a cocktail [trifluoroacetic acid (TFA)/thioanisole/ethanedithiol/anisole (90:5:3:2, v/v/v/v)] at RT for ~3 h. Later, the peptide precipitation from the TFA was initiated by its dropwise addition to freezing diethyl ether. Precipitated peptide was ultracentrifuged at 7000 rpm for 10 min, and the acquired peptide was further rinsed with diethyl ether to remove all the traces of TFA. The dried peptide was dissolved in glacial acetic acid (20%) and then lyophilized (FDUT-12003, Operon, Korea). Similar practice was repeated for the solid phase synthesis of B-Anti G. Both peptides were characterized using Waters HPLC Q-TOF mass instrument (USA). G-Anti G: Expected mass, 804.10 Da; Apparent mass obtained, 804.35 Da $(M + 3H)^{3+}$. B-Anti G: Expected mass, 1236 Da; Apparent mass obtained, 1239 Da $(M + 2H)^{2+}$.

1.4.2. Glioma Specific Toxicity of the Chimeric Peptides

Concisely, C6 cells (10,000/well) were grown in 96-well platforms (Millipore). Subsequently, used media was exchanged with fresh culture medium, DMEM containing the peptides, G-Anti G, B-Anti G, DOX, and TMZ at increasing concentrations and the cells were additionally incubated upto 24 h. Next, cells were incubated with complete media containing MTT reagent (20μL, 5 mg/mL in PBS) and incubated for another 4 h. Then the complete media was removed and purple-colored formazan formed was dissolved in DMSO, and the optical absorption of the solution was recorded at 570 nm by using a microtiter plate reader (Biotech Synergy H1, Finland) and the percentage cell viability was determined in comparison to the control untreated group.

1.4.3. Fabrication and Bio-physical Characterization of Self-Assembled Peptide Based Nanostructures

At first, we explored the self-assembling and nanostructure forming potential of the synthesized amphipathic chimeric peptide drugs. Dynamic light scattering (DLS) was employed to discover the self-assembling process and in solution dimensions of the fabricated peptide-based self-assemblies (Figure S1, supporting information). DLS data revealed that the chimeric peptides (G-Anti G and B-Anti G) could self-assemble in hexa-fluoro-2-propanol (HFIP)-water (1:19 v/v) to form discrete structures with G-Anti G having an average hydrodynamic diameter of 165 ± 2 nm (PDI: 0.2) and B-Anti G having an average diameter of 146 ± 0.3 nm (PDI: 0.1) at a very modest concentration (1 mg/mL).

1.4.4. NIR-808 Laser Induced Temperature Rise by PINPs

Briefly, the photothermal effect (i.e., NIR-808 laser induced temperature rise) of the PNPs and PINPs (0-500 μg/mL), at various concentrations were determined in a quartz cuvette. For this, the samples were irradiated (15 min) by using a NIR-808 laser (from Aimil Technologies). DI and ICG solution (at an equivalent concentration of 21 μg/mL) was taken as a standard reference for assessment. During the entire irradiation span, a thermocouple was remained dipped into the cuvette and the temperature of the

nanoparticle's aqueous solution was monitored and recorded every single min by a digital thermometer.

1.4.5. Determination of Cellular Internalization Ability of PNPs in C6 Glioma Cells

For assessing the cellular uptake/internalization efficiency of the PNPs in C6 cells, PNPs were packed with the fluorescent dye, Rho. Cellular internalization of Rho loaded PNPs as compared to free Rho was probed using a confocal microscope (LSM880 Carl Zeiss System, Germany). Concisely, C6 cells (50,000/well in 6-well dishes) were seeded and nurtured in complete DMEM medium. Then, Rho loaded PNPs (100 μg/mL), and Rho solutions were added to the respected wells and the sample was again incubated for 2 h. Subsequently, wells were rinsed twice with PBS to get rid of the non-internalized PNPs. Trailed to washing, cell nuclei were stained with Hoechst (for 5 min), followed by another PBS washing and addition of 1 mL of phenol free media. Lastly, live imaging of C6 cells was carried out under a confocal microscope (405 nm/Hoechst and 488 nm/Rho) [1,2].

1.4.6. Scratch Assay of PINPs Determined in C6 Glioma Cells

Scratch wound healing assay of PNPs, PINPs, and ICG (100 μg/mL) were performed in C6 glioma cells. In short, C6 cells (50,000/well) were grown in 6-well platforms for 24 h or till the time when the cells had touched ~90% confluency (monolayer). At this stage, a straight scrape was made over the middle of each well by a 200 μL pipette tip. Subsequent to scraping, the wells were mildly washed with PBS and phenol free media to remove all the detached cells. Thereafter, cells were treated with different formulations (PNPs, PINPs, and ICG) for 1 h. After 1 h of incubation span, media from each well was removed and the wells were rinsed with PBS to remove extracellular non-internalized formulations. Next, cells were irradiated with NIR-808 (10 min) followed by live imaging the cells which migrated towards the cell free zone in the scrape at 0 and 24 h, using a brightfield inverted microscope (IX71; Olympus Corp., Tokyo, Japan). Cell migration was examined by measuring the dimension of the gap distance using Adobe Photoshop CC software. Each experiment was done in quadruplicates [1,2].

1.4.7. Localized Chemo-phototoxicity of PINPs Determined in C6 Cells

Next, we quantified the synergistic chemo-phototoxicity of PINPs (10 μg/mL) in comparison to other individual moieties [PNPs (chemotherapy) and ICG (phototherapy)] in C6 cells. In short, C6 cells were nurtured in 96-well platforms in quadruplicate for 24 h. Following day cells were treated with different formulations for 30 min. Thereafter, cells were rinsed twice with PBS to get rid of extracellular non-internalized formulations, and wells were replenished with 200 μL of phenol free media. For synergistic chemo-phototoxicity, corresponding wells were irradiated with NIR-808 laser (10 min). Cells were incubated for another 24 h and were evaluated by standard MTT assay as described previously [1,2].

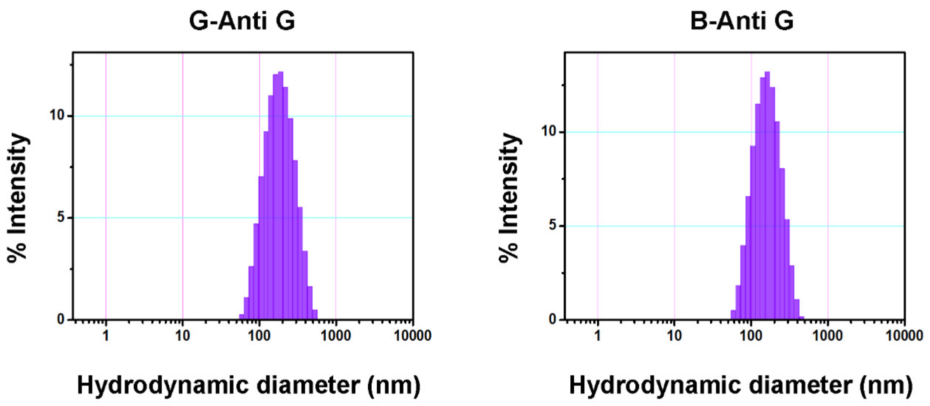

Figure S1. Size distribution profile of self-assembled peptide nanostructures determined in a mixture of HFIP-water at a ratio of 1:19 *v/v*.

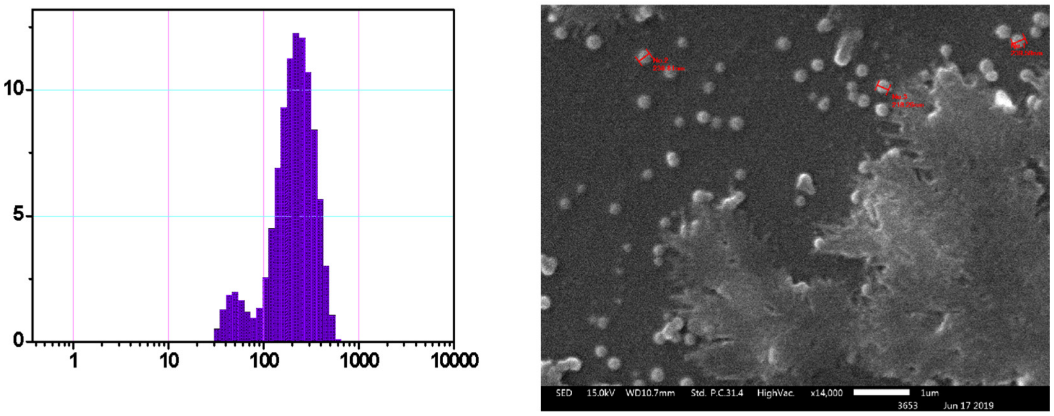

Figure S2. Synthesis and characterization of multicomponent peptide based co-assembled nanostructures (PNPs) in HFIP-water (1:19 *v*/*v*) mixture. (A) Size distribution profile. (B) Scanning electron microscopic image of G-Anti G and B-Anti G co-assembled nanoparticles.

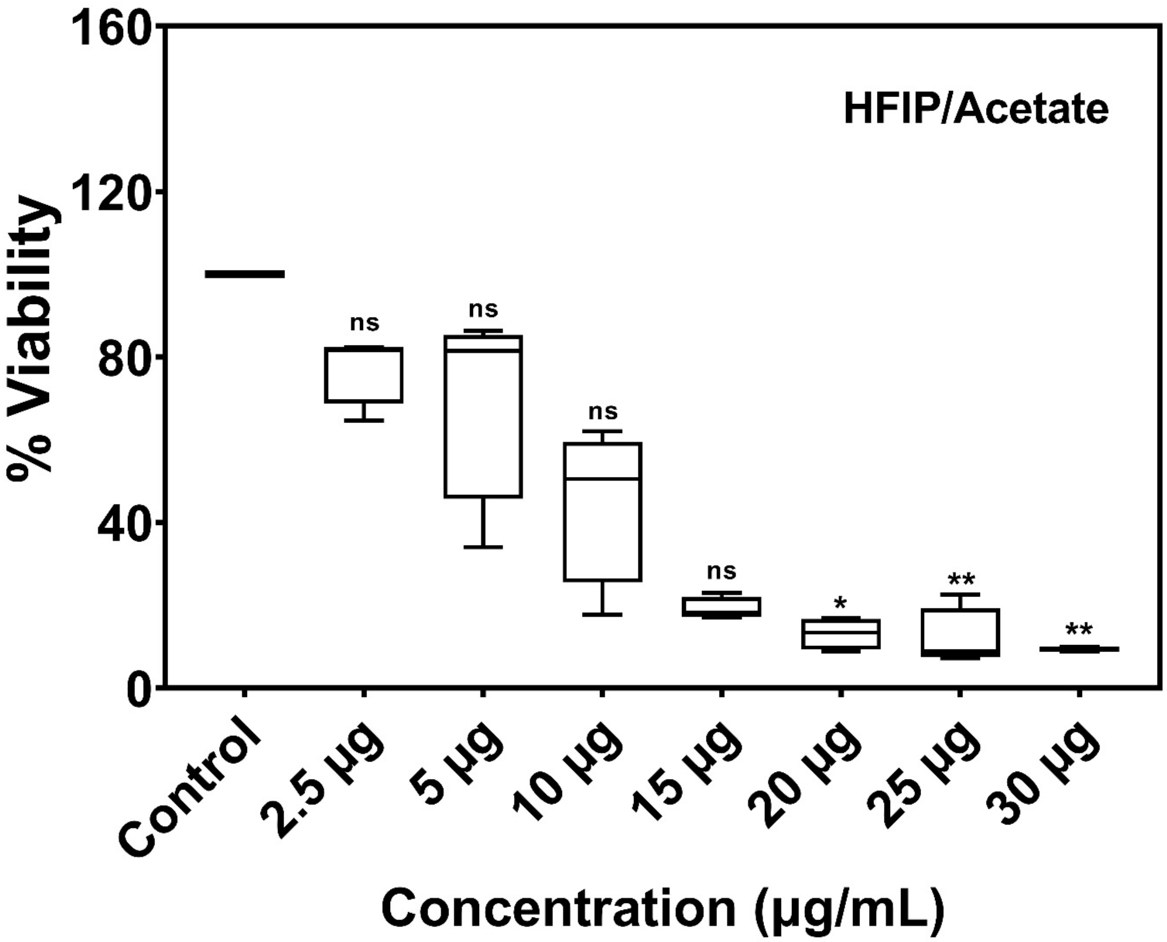

Figure S3. Dose-response experiment i.e., cell viability of C6 glioma cells determined after 24 h of incubation with increased concentrations of PNPs (assembled in HFIP-acetate mixture). Results are presented as median & quartiles (min to max) in quadruplicate. The bar inside the box represents the median. Statistical analysis was carried out using Kruskal–Wallis test followed by Dunn’s post hoc test. Asterisk (*) refers to the statistically significant difference between the control untreated cells vs other concentrations. (*$p < 0.05$; **$p < 0.01$; ns = non-significant).

References

[1] Dube, T.; Kumar, N.; Bishnoi, M.; Panda, J. J., Dual blood–brain barrier–glioma targeting peptide–poly(levodopamine) hybrid nanoplatforms as potential near infrared phototheranostic agents in glioblastoma. Bioconjugate Chemistry 2021, 32 (9), 2014-2031.

[2] Dube, T.; Kompella, U. B.; Panda, J. J., Near infrared triggered chemo-PTT-PDT effect mediated by glioma directed twin functional-chimeric peptide-decorated gold nanoroses. Journal of Photochemistry and Photobiology B: Biology 2022, 228, 112407.
